# Mutation profile and immunoscore signature in thymic carcinomas: An exploratory study and review of the literature

**DOI:** 10.1111/1759-7714.13765

**Published:** 2021-03-11

**Authors:** Rosanna Asselta, Luca Di Tommaso, Matteo Perrino, Annarita Destro, Laura Giordano, Giulia Cardamone, Luca Rubino, Armando Santoro, Stefano Duga, Paolo Andrea Zucali

**Affiliations:** ^1^ Department of Biomedical Sciences Humanitas University Milan Italy; ^2^ Humanitas Clinical and Research Center IRCCS Milan Italy; ^3^ Unit of Pathology IRCCS, Humanitas Clinical and Research Center Milan Italy; ^4^ Department of Oncology IRCCS, Humanitas Clinical and Research Center Milan Italy; ^5^ Statistic Unit IRCCS, Humanitas Clinical and Research Center Milan Italy

**Keywords:** Germline mutation, immunoscore, next‐generation sequencing, somatic mutation, thymic carcinoma

## Abstract

**Background:**

Significant efforts have been made to investigate the molecular pathways involved in thymic carcinogenesis. However, genetic findings have still not impacted clinical practice. The aim of this exploratory trial was to evaluate the immunoscore and molecular profile of a series of thymic carcinomas (TCs), correlating this data with clinical outcome.

**Methods:**

Formalin‐fixed, paraffin‐embedded (FFPE) TC tissues were retrieved from our center archive. The immunoscore was evaluated according to Angell and Gallon. DNA was extracted from FFPE tumor samples and, when available, from adjacent histologically normal tissues. Next‐generation sequencing (NGS) was performed targeting hotspot regions of 50 oncogenes and tumor suppressor genes.

**Results:**

A series of 15 TCs were analyzed. After a median follow‐up of 82.4 months, the median overall survival was 104.7 months. The immunoscore was >2 in 5/15 patients (33%). Among the investigated genes, absence of mutations was observed in 5/15 patients (33%), whereas three variants in 1/15 (6%) patient, two variants in 4/15 (26%) patients, and one variant in 5/15 patients (33%) were found. The most recurrently mutated genes were *FGFR3* (five mutations) and *CDKN2A* (three mutations, two of which were nonsense). Patients with *CDKN2A* loss showed a statistically significantly worse survival (*P* = 0.0013), whereas patients with *FGFR3* mutations showed a statistically significantly better survival (*P* = 0.048).

**Conclusions:**

This study adds data to the few existing reports on the mutational landscape of TCs, providing the first comprehensive analysis to date. Here, we confirm the low rate of mutations in TCs and suggest *FGFR3* and *CDKN2A* mutations as intriguing potential therapeutic targets.

## Graphical abstract

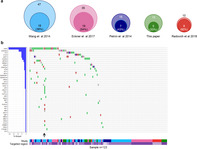

Immunoscore, molecular profile, and their correlation with the clinical outcomes of a series of 15 thymic carcinomas were evaluated. We confirmed the low rate of mutations in thymic carcinomas and suggest *FGFR3* and *CDKN2A* mutations as intriguing potential therapeutic targets

## Introduction

Thymic epithelial tumors (TETs) are still considered rare tumors, with an incidence of 0.15 cases out of 100 000 per year which account for only for 0.2%–1.5% of all malignancies.^1^ Thymic carcinomas (TCs) are more rare, representing approximately 15% of all TETs.^2^ The rarity of these tumors has precluded the development of large phase II and III clinical trials for many years, thus delaying the investigation of new drug therapies. Moreover, the etiology of TETs is unknown. Over the past few years, significant efforts have been made to investigate the underlying molecular pathways involved in thymic carcinogenesis. Consequently, molecular analysis has facilitated the identification of important oncogenes (*EGFR*, *HER2*, *IGF1R*, *KIT*, *KRAS*, and *BCL2*), tumor suppressor genes (*TP53*, *CDKN2A*), chromosomal aberrations (LOH3p,6p,6q,7p,8p), angiogenic factors (VEGF), and tumor invasion factors (matrix metalloproteinases and tissue inhibitor of metalloproteinases) as major contributors to disease development.^3^ Subsequent studies reported, by using different platforms, the association of distinct molecular clusters with different histological subtypes.^4^, ^5^, ^6^, ^7^, ^8^ In particular, TCs have been found to display more chromosomal losses and gains and occasionally harbor somatic mutations in *KIT*.^5^ Despite the limited number of samples analyzed in these studies, unique molecular changes in TETs have been found, such as dysregulation of antiapoptotic genes and mutations in genes involved in histone modification, DNA methylation, and chromatin remodeling.^9^ More recently, Radovich and colleagues performed a multiplatform, comprehensive analysis of TETs as part of The Cancer Genome Atlas (TCGA) project to uncover the integrated genomic landscape of these rare tumors.^10^ They found that thymomas have the lowest mutational burden among adult cancers. However, the number of TCs analyzed was limited (only 10 cases) and these genetic findings do not significantly affect the clinical practice. Therefore, improving our understanding of the molecular biology of these rare tumors remains a key challenge.

The aim of this study was to evaluate the molecular profile and the immunoscore of a series of 15 TCs, and to correlate the identified molecular profiles with clinical characteristics and patient survival data.

## Methods

### Tissue samples

Formalin‐fixed, paraffin‐embedded (FFPE) TC (*n* = 15 patients; six squamous cell, four basaloid, three lymphoepithelioma‐like, and two adenocarcinomas) tissues were retrieved from the archive of the Department of Pathology, Humanitas Clinical and Research Center of Rozzano (Milan, Italy). The tumors had been diagnosed and subtyped according to the WHO classification between 2000 and 2010.^11^

### Clinical data

The clinical data collection included patient age, sex, tumor anatomic location, WHO histological classification, Masaoka Staging, date and kind of treatments, vital status, date of death, and date of last contact. The study was conducted in accordance with the ethical principles of the Declaration of Helsinki and local regulations and was approved by the institutional Ethic Committee. This trial was registered at www.clinicaltrials.com (NCT00965627).

### Immunohistochemical analyses

Immunohistochemical analysis was performed using an automated staining system (Discovery XT, Ventana Medical Systems, AZ, USA) according to the manufacturer's instructions. Antibodies, sources, clones and dilutions are detailed in Table S1. Staining was evaluated by an expert pathologist (LDT) and reported as positive/negative, percentage of immuoreactive cells or a score according to the following criteria. For CD5, cKIT, chromogranin and synaptophisin cases were considered positive if they showed an unequivocal immunoreactivity in ≥5% of tumor cells; for PD‐L1, cases were considered positive if the percentage of tumor or inflammatory cells showed membranous staining ≥1%; Ki67 was quantified as the percentage of tumor cells which showed nuclear staining. The immunoscore was evaluated according to Angell and Gallon^12^ Briefly, once evaluated the median immune CD3+ and CD8+ cells density within the tumor and at its margins in the whole series, each patient received a binary score (0 low; 1 high) for each immune type and tumor region. The sum of these four values represented the immunoscore.

### DNA extractions, next‐generation sequencing (NGS) and bioinformatics analyses, validation of newly identified variants

Tumor samples were obtained from 15 patients with TC, and histologically normal tissues from adjacent resected tissues were obtained from a subset of eight patients. DNA extraction from FFPE samples; DNA quantification and quality assessment were performed by qPCR, using the KAPA hgDNA Quantification and QC Kit (Kapa Biosystems; Wilmington, MA, USA). The generated library targets hotspot regions of 50 oncogenes and tumor suppressor genes (listed in Table S2). Details of NGS, bioinformatics analyses and validation of newly identified variants^13^, ^14^, ^15^, ^16^, ^17^, ^18^, ^19^, ^20^, ^21^ are provided in Supporting Information.

### Statistical analysis

Data were described as number and proportion or as median and range. Fisher's exact test was used for association of molecular subtypes with the immunohistochemistry score. Overall survival was defined as time between diagnosis and death or the last time the patient was known to be alive. The association of each molecular subtype with survival outcomes was estimated using Kaplan‐Meier plots and log‐rank tests. Cox proportional hazards regression analysis was used to evaluate continuous prognostic factors associated with survival outcomes. Statistical significance was set at *P* < 0.05, and all tests were two‐tailed. All analyses were performed using SAS version 9.4.

## Results

### Clinical outcomes and demographics

Table 1 summarizes the patient characteristics. The median age of the patients was 64 years (33–77 years). With a median follow‐up of 82.4 months, the median overall survival was 104.7 months. At diagnosis, the disease was localized (stage I–II) in six patients (40%) and locally advanced or metastatic (stage III–IV) in eight patients (53%). A total of 11 patients (78.5%) received radical surgery (R0) on a primary tumor, whereas three (14.2%) were treated with microscopically (R1: two patients) or macroscopically (R2: one patient) not radical surgery. Six patients (20%) received at least one line of chemotherapy and three were treated with more than one line of chemotherapy. Patients with localized disease at diagnosis showed a statistically significantly longer survival (*P* = 0.005).

**Table 1:** Patient characteristics

Characteristics	No/median	% / range
**Age (years)**	64	33–77
**Sex**		
Male/female	12/3	80/20
**Histological type**		
Thymic carcinoma	15	100
Squamous/basaloid/lymphoepithelioma/adenocarcinoma	6/4/3/2	40/27/20/13
**Stage of disease** ^†^		
Stage I‐II‐III‐IVa‐IVb‐Uk	2/4/3/0/5/1	13/27/20/0/33/7
**Type of treatment**		
Surgery primary tumor		
Yes/No/Uk	14/0/1	93/0/7
Adjuvant radiotherapy		
Yes/No/UK	5/5/5	33/33/33
Chemotherapy		
<2 lines/≥2 lines/no/Uk	3/3/4/5	20/20/27/33

No, number; Uk, unknown.

† Masaoka stage.

### Immunohistochemical analysis and immunoscore

Morphological and phenotypical features are summarized in Table 2. Two lesions were characterized by mild cytological atypia and low Ki67 values, consistent with low grade TC; all the remaining cases showed a severe atypia and/or high Ki67 values in keeping with high grade TC. A positive expression of CD5, neuroendocrine markers, and cKIT was observed in 12 (80%), eight (53%), and 12 (80%) patients, respectively. The PD‐L1 was positive in eight patients (53%). In particular, PD‐L1 expression was observed in tumor cells in seven patients, whereas in the tumor microenvironment in only one patient. The immunoscore was >2 in five patients (33%). None of the biomarker results were statistically significantly related either to stage of disease, or to survival outcomes.

**Table 2:** Clinical and pathological features of the analyzed thymic carcinoma (TC) patients

Patient	Histotype	Ki67 (%)	CD5	cKIT	NE‐markers	PD‐L1	Immunoscore
1	Squamous	40	−	−	+	−	1
2	Squamous	25	+	+	−	+,t	4
3	Lymphoepithelioma‐like	20	+	+	−	+,t	2
4	Squamous	30	+	+	+	+,t	2
5	Lymphoepithelioma‐like	30	+	+	+	+,i	2
6	Lymphoepithelioma‐like	20	+	+	+	+,t	2
7	Squamous	20	+	+	+	−	0
8	Basaloid	60	+	+	−	+,t	2
9	*Adenocarcinoma (adenoid cystic)*	5	−	−	−	−	nv
10	Adenocarcinoma (mucinous)	20	−	−	−	−	3
11	Squamous	10	+	+	−	−	4
12	Basaloid	30	+	+	−	+,t	nv
13	Basaloid	20	+	+	+	+,t	4
14	*Basaloid*	10	+	+	+	−	0
15	Squamous	30	+	+	+	−	4

NE, neuroendocrine; the immunoscore has been evaluated in keeping with reference 22 and ranges between 0 and 4 (for a detailed description see methods); +, positive; −, negative; t: tumoral cells; i: inflammatory cells; in *italic* low grade lesions.

### Mutational analysis of TCs

We performed NGS of hotspot regions of a total of 50 onco‐ and tumor‐suppressor genes in 15 TC samples, of which eight were matched with normal adjacent tissue. The overall mean coverage was 1524X ± 679X, with a uniform distribution of reads across amplicons. On average, NGS identified 18 variants per sample. Variants with <4% of coverage as well as common SNPs were removed, leaving a total of 16 high‐quality variants. These variants were searched in somatic mutations databases (Cosmic, cBioPortal) as well as in the germline mutation repository GnomAD; their possible deleteriousness was investigated in silico by using four prediction software for missense and four programs for splicing variant evaluation. Novel variants were confirmed by Sanger sequencing (if they showed a percentage of mutant reads between 15% and 40%), by allele‐specific assays (if they showed a percentage of mutant reads <15%), or by a PCR amplification followed by agarose gel electrophoresis (in the case of the 40‐nucleotide‐long deletion). The results of these analyses are reported in Table 3.

**Table 3:** Mutational burden of thymic carcinomas (TCs) of the present series

Patient	Identified variant/s	Gene	% of reads	Present in databases^†^	Predicted deleteriousness^‡^	Control tissue
1	p.Phe858Leu	*ATM*	54.2	Cosmic,cBioPortal,GnomAD	ND,ND,D,D	n.a.
	p.Gly13Arg	*NRAS*	40.9	Cosmic,cBioPortal	D,D,D,D	
2	No variants				‐	n.a.
3	p.Gly116Glu^§^	*CDKN2A*	4.8	Cosmic	ND,ND,D,D	n.a.
4	No variants				‐	n.a.
5	p.Glu88Ter	*CDKN2A*	66.5	Cosmic,cBioPortal	‐	n.a.
6	**p.Pro772Arg**	*FGFR3*	11	Novel	D,D,D,D	n.a.
	p.Gly533Arg	*SRC*	6	GnomAD	D,D,D,D	
7	**IVS4 + 24C > G or p.Thr175Ser** ^¶^	*SMARCB1*	48.2	Novel	No impact on splicing ND,ND,ND,D	n.a.
8	p.Arg80Ter	*CDKN2A*	85.2	Cosmic,cBioPortal (thymic carcinoma)	‐	Absent in the control tissue
9	No variants					
10	**Out‐of‐frame deletion of 40 nt (g.chr5:112174661)**	*APC*	100	Novel	‐	Absent in the control tissue
	IVS10 + 4A > G	*KIT*	44	GnomAD	DYS,0.39,0.72,0.83	Detected also in the control tissue (47% reads)
11	p.Ser249Cys	*FGFR3*	9.6	Cosmic (thymic carcinoma), cBioPortal	D,D,D,D	Absent in the control tissue
	p.Phe384Leu or p.Phe386Leu	*FGFR3*	46.2	Cosmic,cBioPortal,GnomAD	ND,ND,D,D	Detected also in the control tissue (51.6% reads)
	p.Met362Thr	*MET*	47.3	Cosmic,GnomAD	D,ND,ND,D	Detected also in the control tissue (51.6% reads)
12	p.Ser249Cys	*FGFR3*	24.3	Cosmic (thymic carcinoma), cBioPortal	D,D,D,D	Absent in the control tissue
	p.Arg374Gln	*SMARCB1*	40.8	Cosmic,cBioPortal	D,D,D,D	Absent in the control tissue
13	**p.Tyr381His**	*FGFR3*	34.4	Novel	D,D,D,D	Detected also in the control tissue (47.6% reads)
14	No variants					
15	No variants					

Novel‐described mutations are indicated in bold.

† The presence of all identified variants was checked in the Cosmic, cBioPortal, and the GnomAD repositories (all databases accessed in January 2019). Putative germline variants already described in the GnomAD database were reported only if they showed a minor allele frequency < 1% in the general population.

‡ In‐silico predictions were performed for all missense and splicing variants. The programs used for missense predictions were: SIFT, PolyPhen2, MutationTaster, and LRT. D (damaging) and ND (not damaging) scores are reported in order for the listed programs. The algorithms used for splicing variant predictions were: HSF, NetGene2, SSPNN, and ADA. Scores are ordered as follows: for splice‐site prediction using HSF, signals above 65 are considered as strong splice sites; if the wild‐type score is above the threshold, and the score variation between the wild‐type and mutant sequence is higher than 10%, the mutation is considered to break the splice site and is indicated as disruptive (DYS). For NetGene2 and SSPNN, scores are between 0 and 1; higher scores imply a higher confidence of true splice site. As for ADA predictions, scores above 0.7 was used to define a variant as splice‐altering.

§ This missense substitution is mapping in the alternative transcript of *CDKN2A* (RefSeq CDKN2A_ENST00000361570), characterized by the presence of an additional upstream exon. This transcript is known to code for a protein isoform, and is able to interact with p53.

¶ This can be regarded either as a splice variant or as a missense substitution (when it involves the alternative transcript of the *SMARCB1* gene; RefSeq SMARCB1_ENST00000344921).

Aa, amino acid; n.a., not available; nt, nucleotide; TCs, thymic carcinomas.

One third of patients did not show any mutation in the investigated genes, whereas one patient was a carrier of three variants, four patients were carriers of two variants, and the remaining five patients each showed one variant. Five out of the 16 variants were clear somatic mutations, whereas four were also detected in the control tissue and possibly represented germline variants. The most recurrently mutated genes were *FGFR3* (five mutations) and *CDKN2A* (three mutations, two of which were nonsense). The only recurrent mutation was the p.Ser249Cys missense substitution in *FGFR3*, found in two patients, and already reported in the Cosmic database as being associated with TCs.

Patients with loss of *CDKN2A* showed a statistically significantly worse survival (four years OS NEG: 83.3%, POS: 0%; *P* = 0.0013), whereas patients with *FGFR3* mutations showed a statistically significantly better survival (four years OS NEG: 54.6%, POS:100%; *P* = 0.048).

No statistically significant correlation was observed between the mutations observed, or between the single mutations, and the immunoscore or the stage of the disease; no correlation was found between the number of mutations and survival.

## Discussion

The results of our study are consistent with the literature data confirming a low rate of mutations in TC.^5^ In fact, one third of our patients did not show any mutation in the investigated genes, whereas one patient was a carrier of three variants, four patients were carriers of two variants, and the remaining five patients only showed one variant each. Although the panel of genes used was limited (only 50), the sensitivity of our test was adequate, considering that the overall mean coverage was more than 1500X. Certainly, the analysis of a greater number of tumors and/or genes may have highlighted other significantly mutated genes or additional clinically, or biologically relevant mutations in other genes outside our panel.

Histology of TC has been historically separated into low‐ and high‐grade lesions.^23^ Even if the clinical impact of this grading has been questioned, the results of the present study suggest that it may be indicative of tumor biology. Indeed, low‐grade lesions were characterized by the absence of mutations; high‐grade lesions without mutations showed the highest immunoscore level; finally, among high‐grade lesion with mutations, those with *FGFR3* mutations showed a proliferative fraction (as evaluated by Ki67) lower than the remaining cases, as opposed to those with *CDKN2A*, which had a higher proliferative fraction.

In our 15 tumor samples, the most recurrently mutated genes were *FGFR3* and *CDKN2A* and the only recurrent mutation was p.Ser249Cys in *FGFR3*. Despite reports in the literature suggesting that mutations of *TP53* are frequent in TCs (approximately 25%), we did not observe point mutations in this gene. Interestingly, null mutations in *APC* (frameshift) and *CDKN2A* (two nonsense) showed a percentage of reads corresponding to the mutated allele higher than expected for a heterozygous somatic mutation (Table 3), suggesting the possible combination, in trans, with a gene deletion. The same higher‐than‐expected percentages of mutated allele were found for *CDKN2A* by Wang *et al*.^9^ and Enkner *et al*.^24^; moreover, this is compatible with the identification of deletions involving the *CDKN2A* genomic regions in TCs.^7^, ^10^

**Figure 1:**
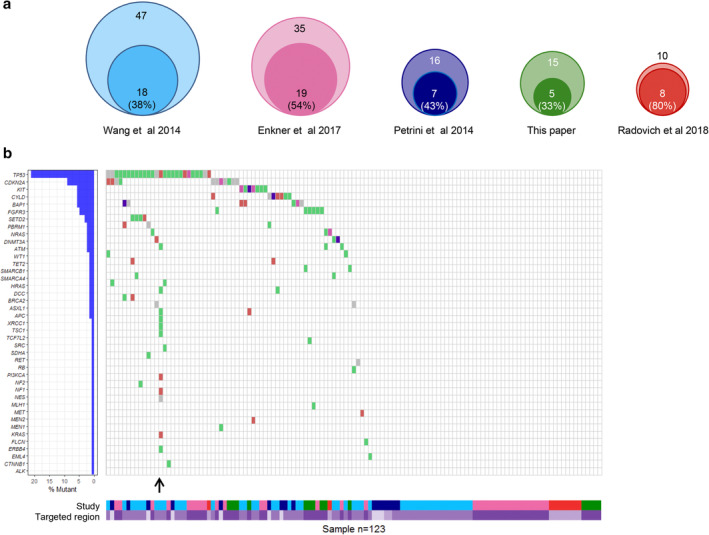
Mutational landscape of thymic carcinomas (TCs): A literature review. (**a**) The Venn diagrams (drawn as proportional objects) represent each analyzed study^7^, ^9^, ^10^, ^24^: The outer circle represents the analyzed patient cohort (the total number of sequenced cases is indicated at the top), the inner circle corresponds to the subset of patients in whom no mutations were identified (the total number of patients as well as the corresponding percentage are reported) (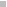) Nonsense mutation (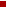) Frame Shift Ins (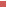) Frame Shift Del (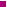) In Frame Ins (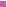) In Frame Del (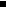) Nonstop mutation (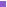) Translation start site (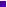) Splice site (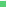) Missense mutation (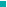) 5'Flank (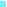) 3'Flank (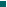) 5'UTR (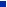) 3'UTR (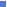) RNA (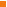) Intron (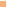) IGR (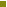) Silent. (**b**) The waterfall plot describes the main results of the analyzed studies. On the left, the plot reports the list of the 41 mutated genes in TCs, together with the corresponding percentage of individuals carrying a mutation (in percentage calculations, only mutated individuals have been considered). The right plot shows the types of mutations in each tumor sample. The only sample showing two deleterious mutations in the same gene (*ATM*) is indicated by an arrow. The lower part of the figure shows the targeted region investigated in each sample and indicates the relevant study (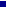) Petrini et al., 2014 (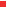) Radovich et al., 2018 (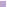) Exome (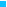) Wang et al., 2014 (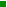) This article (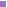) 197 genes (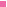) Enkner et al., 2014 (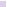) Exome + 197 genes (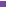) 50 genes.

Figure 1 summarizes our data and that in the literature evaluating gene mutations in wide gene sets (at least 50) in different series of TCs. All the series of TCs explored were small (range 10–47 cases)^7^, ^9^, ^10^, ^24^ and showed a low rate of mutations (percentage of tumors with at least one mutation ranging from 20% to 67%). Out of a total of 41 genes found mutated in TCs, the most recurrently mutated were *TP53* (39% of all mutated tumors; 21% of all analyzed TCs) and *CDKN2A* (17% and 9%), confirming that TCs are a molecularly heterogeneous group of tumors driven by a limited number of genomic events. Of the 66 mutated tumors, 34 showed just one mutation, and the others were characterized by the presence of two to four mutations; one sample showed an exceptional number of 12 mutations, accordingly with the presence of two deleterious variants in the *ATM* gene. We are aware that this mutational landscape derives from heterogeneous studies, which analyzed different – although overlapping – target regions.

As observed in previous studies, patients carrying *CDKN2A* mutations (three out of 15 patients, 20%) showed a statistically significantly worse survival.^25^, ^26^ The *CDKN2A* gene is located on chromosome 9p21.3 and encodes p16 (INK4a) and p14_ARF_. The p16 protein is a negative regulator of cell cycle progression which blocks cyclin‐dependent kinase 4 (CDK4) and 6 (CDK6), whereas the p14_ARF_ activates the *TP53* tumor suppressor. A *CDKN2A* inactivating mutation may lead to activation of cyclin dependent kinases, thus permitting an inappropriate progression through the cell cycle and promoting uncontrolled tumor cell proliferation. Currently, several inhibitors for these kinases are being investigated in clinical trials for various malignancies and might constitute a therapeutic option. In two phase II trials, it has been reported that milciclib (PHA‐848125AC), a potent inhibitor of CDKs, is safe, well‐tolerated, and achieved disease stabilization in a majority of patients with TC and B3 thymoma.^27^

The patients in our study carrying *FGFR3* mutations (4/15 patients, 26.6%) showed a statistically significantly better survival. Enkner *et al*. observed an *FGFR3* missense mutation in two out of 35 TCs,^24^ while no *FGFR3* mutations were reported in 42 TCs screened with a 197‐gene panel including *FGFR3*.^9^ *FGFR* genes are deregulated in solid tumors by amplification, translocation or mutation and mutations and fusions in *FGFR2/3* can lead to constitutive FGFR signaling that may contribute to carcinogenesis.^22^ *FGFR3* mutations have been found to be particularly frequent in bladder cancer (20%), where the results showed an association with early stage, low grade, and better survival.^28^ Several FGFR inhibitors are currently under investigation in clinical trials. In a phase II trial, erdafitinib, a potent tyrosine kinase inhibitor of FGFR 1–4, was found to be associated with an objective tumor response in 40% of previously treated patients who had locally advanced and unresectable or metastatic urothelial carcinoma with at least one *FGFR3* mutation or *FGFR2/3* fusion.^29^ In a recent phase II trial, lenvatinib, a potent inhibitor of receptor tyrosine kinases, targeting vascular endothelial growth factor receptors (VEGFR1‐3), FGFR1‐4, KIT, and RET, showed clinical efficacy in 42 patients with advanced or metastatic TC, with an overall response rate of 38%, disease control rate of 95.2%, and median duration of response of 11.6 months.^30^ Thus, inhibition of FGFR3 might represent an intriguing novel target in a subset of TCs. Moreover, patients with urothelial carcinoma carrying *FGFR* alterations may be less likely to have a response to immunotherapy than are those without such alterations.^29^, ^31^ Considering that pembrolizumab showed an interesting activity in patients with TC,^32^, ^33^ it may represent a potential predictive biomarker.

In our series, a *KIT* putative splicing variant (GnomAD allelic frequency 3.98 × 10^−6^) was detected in one patient. *KIT* mutations are a therapeutic target for kinase inhibitors such as imatinib, and represent the only known druggable targets in TCs, as evidenced by few but encouraging case reports.^24^ However, the data in the literature confirm a low rate of *KIT* mutations in TCs.^7^, ^9^, ^24^

Only one of the patients in our study harbored a *NRAS* mutation. At present, the RAS oncogenes are still not druggable targets.

The immunoscore was >2 in five patients (33%), whereas the PD‐L1 expression by immunohistochemistry was positive in eight patients (53%), which was consistent with previous data in the literature.^24^, ^32^, ^33^ No statistically significant correlation between immunoscore and PD‐L1 expression, mutations, stage of disease, or survival outcomes was observed. An absence of survival difference between positive and negative PD‐L1 expression in patients with TC has also been described in other studies.^24^, ^34^ On the other hand, considering the correlation of PD‐L1 expression by tumor cells with the likelihood of response to anti‐PD‐1/PD‐L1 therapy, immune checkpoint inhibitors might be an active treatment option for unresectable or relapsed TCs. In fact, recently, two phase II trials showed promising activity of pembrolizumab as monotherapy in TCs and this activity correlated with PD‐L1 expression.^32^, ^33^

The major limitations of our study are: (i) the use of archive FFPE samples, for which DNA extraction may suffer from the degradation of nucleic acids by formalin fixation as well as formalin‐induced sequence artifacts; however, we carefully quality‐checked the extracted DNA with the deep sequencing step (given that the artifacts are random, they become less apparent as sequencing depth increases); (ii) the small number of analyzed cases; and (iii) the limited number of genes analyzed, precluding therefore larger and deeper analyses to discover other significantly mutated genes or additional clinically or biologically relevant mutations. However, our study confirms the low rate of mutations in TC compared with other solid tumors and suggests *FGFR3* and *CDKN2A* mutations as intriguing potential therapeutic targets. A better understanding of the molecular architecture of thymic neoplasms may potentially impact on disease classification, targeted therapeutic decision‐making, and the design of future clinical trials.

## Disclosure

No authors report any conflict of interest

## Supporting information


**Appendix S1** Supporting Information

**Table S1** Antibodies, sources, clones and dilutions for immunohistochemistry

**Table S2** List of 50 oncogenes and tumor suppressor genes
