# Supplementary material for: Mutation profile and immunoscore signature in thymic carcinomas: An exploratory study and review of the literature
Source: Thorac Cancer. 2021 Mar 11;12(9):1271–8. doi: 10.1111/1759-7714.13765 (PMC8088947; doi:10.1111/1759-7714.13765)
Supplement: Supplementary file 1 — Appendix S1 Supporting Information [file TCA-12-1271-s002.doc]

**SUPPORTING INFORMATION**

**DNA extractions**

Tumor samples were obtained from 15 patients with TC, whereas histologically normal tissues from adjacent resected tissues were obtained from a subset of 8 patients. DNA was extracted from FFPE samples using the Maxwell 16 FFPE Tissue LEV DNA Purification Kit and the Maxwell 16 Instrument (Promega, Milan, Italy). DNA quantification and quality assessment were performed by qPCR, using the KAPA hgDNA Quantification and QC Kit (Kapa Biosystems; Wilmington, MA, USA).

**Next-generation sequencing (NGS) and bioinformatics analyses**

The Ion AmpliSeq Cancer Hotspot Panel v2 (Thermo Fisher Scientific; Carlsbad, CA, USA) was used to generate libraries. Briefly, 50 ng of DNA were amplified by PCR using the Panel Primer Pool and the Ion AmpliSeq Library Kit 2.0, according the manufacturer’s recommendations. The library targets hotspot regions of 50 oncogenes and tumor suppressor genes (listed in Supplementary Table 2). Library concentration was determined with the KAPA Library Quantification Kit (Kapa Biosystems). Samples were multiplexed for emulsion PCR and sequencing on an Ion PGM Sequencer (Thermo Fisher Scientific). Sequencing was carried out using the Ion PGM 200 Sequencing Kit (Thermo Fisher Scientific), following the manufacturer's instructions on Ion 316 Chips.

The Torrent Suite version 4.0.2 (Thermo Fisher Scientific) was used to generate sequence alignments with the hg19 human genome reference, to calculate the coverage, and to filter poor signal reads. Variant calling was performed with Ion Torrent Variant Caller 4.0 software, setting a minimum coverage of 20 reads. A threshold of at least 4% mutant reads was set for variant identification.

Identified variants were searched in publicly available repositories for somatic (Cosmic database v87, https://cancer.sanger.ac.uk/cosmic; cBioPortal for Cancer Genomics, http://www.cbioportal.org/) and germline (GnomAD, http://gnomad.broadinstitute.org/) mutations.

Deleteriousness of missense variants, both previously reported and novel ones, was assessed by using five prediction programs: SIFT [13], PolyPhen2 (two algorithms: HumVar and HumDiv) [14], MutationTaster [15], and Likelihood Ratio Test (LRT) [16]. These programs were run through the Variant Effect Predictor (VEP) online tool maintained at the Ensembl resource (https://www.ensembl.org/info/docs/tools/vep/index.html), which takes advantage of the dbNSFP v3.0 databases [17].

Computer-assisted analysis for novel splice-site variants was accomplished by using four prediction tools: Human Splicing Finder (HSF) [18], NetGene2 [19], Splice Site Prediction by Neural Network (SSPNN) [20], and Adaptive Boosting algorithm (ADA) [21].

**Validation of newly identified variants**

Validation of variants was performed either on the same DNA used for targeted NGS or on a newly extracted sample (taken from the adjacent slice of the FFPE block).

Validation of variants showing in the NGS step a percentage of the mutant allele between 15 and 40% was performed by direct Sanger sequencing of the relevant PCR-amplified genomic region (using the BigDye Terminator Cycle Sequencing Ready Reaction Kit v1.1; Thermo Fisher Scientific). Sequencing reactions were run on an ABI-3130XL and analyzed using the Variant Reporter program (Thermo Fisher Scientific).

For variants showing a percentage of mutant reads <15%, we performed allele-specific PCR assays. To this aim, specific PCR primers were designed for each putative variant in such a way that the last nucleotide at the 3' end of the primer was complementary to the identified mutant allele. To further assure the selective amplification of the mutant allele, in each allele-specific primer we also introduced a mismatch a position -3 (with respect to the primer 3’ end).

In the case of a 40-nucleotide-long deletion, the validation was performed by a PCR amplification (bridging the deletion), followed by agarose gel electrophoresis.

All oligonucleotides used in the validation step were purchased from Sigma (St Louis, MO, USA). Their sequences, as well as thermal profiles used for PCRs, are available on request.
