# Supplementary material for: Mutation profile and immunoscore signature in thymic carcinomas: An exploratory study and review of the literature
Source: Thorac Cancer. 2021 Mar 11;12(9):1271–8. doi: 10.1111/1759-7714.13765 (PMC8088947; doi:10.1111/1759-7714.13765)
Supplement: Supplementary file 2 — Table S1 Antibodies, sources, clones and dilutions for immunohistochemistry [file TCA-12-1271-s001.doc]

**Supplementary Table 1.** Antibodies, sources, clones and dilutions for immunohistochemistry

| **Antibody** | **Source** | **Clone** | **Dilution** | **Positivity criteria** |
| --- | --- | --- | --- | --- |
| **CD5** | Ventana (Tucson, AZ, USA) | SP19 | Pre-diluted | ≥5% of tumor cells |
| **c-kit** | Spring Bioscience (Pleasanton, CA, USA) | polyclonal | Pre-diluted | ≥5% of tumor cells |
| **Chromogranin** | Ventana (Tucson, AZ, USA) | LK2H10 | Pre-diluted | ≥5% of tumor cells |
| **Synaptophysin** | Ventana (Tucson, AZ, USA) | SP11 | Pre-diluted | ≥5% of tumor cells |
| **PD-L1** | Spring Bioscience (Pleasanton, CA, USA) | SP142 | Pre-diluted | ≥1% of tumor or inflammatory cells |
| **Ki67** | Ventana (Tucson, AZ, USA) | 30-9 | Pre-diluted | % of tumor cells showing nuclear staining |
| **CD3** | Ventana (Tucson, AZ, USA) | 2GV6 | Pre-diluted | * |
| **CD8** | Ventana (Tucson, AZ, USA) | SP57 | Pre-diluted | * |

*Once evaluated the median immune CD3+ and CD8+ cells density within the tumor and at its margins in the whole series, each patient received a binary score (0 low; 1 high) for each immune type and tumor region. The sum of these four values represented the immunoscore.
